# *Tabernaemontana cameroonensis* nom. nov., a new name for *Domkeocarpa pendula* Markgr. following reassessment of the *Tabernaemontana ventricosa* Hochst. ex A.DC. complex (Gentianales, Apocynaceae)

**DOI:** 10.3897/phytokeys.281.200979

**Published:** 2026-11-06

**Authors:** Marie Claire Veranso-Libalah, Moses B. Libalah, Oreste Yemdji, Andreas Gröger, Bonaventure Sonké, Gudrun Kadereit

**Affiliations:** 1 Senckenberg Research Institute and Natural History Museum, Frankfurt am Main, Frankfurt, D-60325, Germany Senckenberg Research Institute and Natural History Museum Frankfurt am Main Germany; 2 Plant Systematics and Ecology Laboratory, Higher Teachers’ Training College, University of Yaoundé I, Yaoundé, 047, Cameroon Plant Systematics and Ecology Laboratory, Higher Teachers’ Training College, University of Yaoundé I Yaoundé Cameroon; 3 Department of Plant Biology, Faculty of Science, University of Yaoundé I, Yaoundé, 812, Cameroon Department of Plant Biology, Faculty of Science, University of Yaoundé I Yaoundé Cameroon; 4 Botanischer Garten München-Nymphenburg und Botanische Staatssammlung München, Staatliche Naturwissenschaftliche Sammlungen Bayerns, München 80638, Germany Botanischer Garten München-Nymphenburg und Botanische Staatssammlung München, Staatliche Naturwissenschaftliche Sammlungen Bayerns München Germany; 5 International Joint Laboratory DYCOFAC, IRD-UYI-IRGM, Yaoundé, Cameroon International Joint Laboratory DYCOFAC Yaoundé Cameroon; 6 Missouri Botanical Garden, Africa & Madagascar Department, St Louis, MO 63166-0299, USA Missouri Botanical Garden, Africa & Madagascar Department St Louis United States of America; 7 Herbarium et Bibliothèque de Botanique africaine, CP 265, Université Libre de Bruxelles, Boulevard du Triomphe, Brussels B-1050, Belgium Herbarium et Bibliothèque de Botanique africaine, CP 265, Université Libre de Bruxelles Brussels Belgium; 8 Prinzessin Therese von Bayern-Lehrstuhl für Systematik, Biodiversität & Evolution der Pflanzen, Ludwig-Maximilians-Universität München, München, Germany Prinzessin Therese von Bayern-Lehrstuhl für Systematik, Biodiversität & Evolution der Pflanzen, Ludwig-Maximilians-Universität München München Germany

**Keywords:** Africa, Cameroon Volcanic Line, conservation assessment, floral and fruit morphology, montane forests, taxonomy

## Abstract

*Tabernaemontana cameroonensis* Ver.-Lib. & Libalah, **nom. nov**. (Apocynaceae) is proposed as a replacement name for *Domkeocarpa pendula* Markgr. following a taxonomic reassessment of *T. ventricosa* Hochst. ex A.DC. in Central Africa. We show that collections from Cameroon and São Tomé previously identified as *T. ventricosa* correspond to *D. pendula* and are distinct from *T. ventricosa*, Since the epithet ‘*pendula*’ cannot be transferred to *Tabernaemontana*, this taxon is renamed *T. cameroonensis*. The species is characterized by cap-like flower buds, a short, untwisted corolla tube with a pale yellow throat, strongly undulate and asymmetrical corolla lobes, and a compact, deeply bilobed syncarp with a densely verrucose to coarsely granulose surface and irregular longitudinal ridges. It is currently known from around 84 km^2^ of fragmented humid montane forest in western and southwestern Cameroon and São Tomé.

## Introduction

The genus *Tabernaemontana* L. (1753: 210), in Apocynaceae, comprises approximately 120–140 accepted species (WFO 2026) distributed throughout the tropics. Members of *Tabernaemontana* are lianas, shrubs, or small to large trees characterized by abundant white latex, opposite leaves with interpetiolar flaps, and sympetalous flowers with contorted corolla lobes folded inward in bud (Endress et al. 2019). In continental Africa, the genus is represented by 19 species (Jongkind 2022), mainly found in evergreen lowland and montane forests.

Within *Tabernaemontana*, the fruit provide informative characters. These include shape, the degree of divergence or fusion of the mericarp, and ornamentation of the surface (Leeuwenberg 1976). However, fruit are often absent from herbarium specimens.

*Tabernaemontana* is less well-documented in those parts of tropical Africa where botanical exploration remains uneven and extensive forested regions remain under-sampled (Dauby et al. 2016; Sosef et al. 2017).

One problematic species concept is *T. ventricosa* Hochst. ex A.DC (1844: 366). Originally described from Natal, South Africa (De Candolle 1844), the species was later broadly applied to material from eastern, central, and southern Africa (Leeuwenberg 1976) and includes substantial morphological heterogeneity, particularly in the fruit. Specimens from Cameroon possess large, strongly sculptured fruit that differ markedly from those in material from southern and eastern Africa, suggesting that this may represent a different species.

Between 2022 and 2026 in submontane forests near Bandoumkassa in western Cameroon, a distinctive *Tabernaemontana* was collected that is conspecific with *Domkeocarpa pendula* Markgr. and corresponds to material previously placed under *T. ventricosa*. This material is clearly distinct from *T. ventricosa* in southern and eastern Africa.

Here, we rename *D. pendula* as *Tabernaemontana cameroonensis* Ver.-Lib. & Libalah, nom. nov. and we re-evaluate its relationship to *T. ventricosa*.

## Materials and methods

Fieldwork was conducted between 2022 and 2026 in submontane evergreen forests near Bandoumkassa, West Region, Cameroon, at elevations between 800 and 1,550 m a.s.l.

Material of our collections are deposited at BR, BRLU, K, M, and YA.

Additional herbarium specimens were examined at YA and through digitized collections in BR, K, M, MO, WAG, P, and Z (herbarium acronyms according to Thiers 2026).

Particular attention was given to African species of *Tabernaemontana* that are morphologically similar to *T. cameroonensis*, including *T. psorocarpa* (Pierre ex Stapf) Pichon, *T. brachyantha* Stapf (1894: 22), *T. pachysiphon* K. Schum. (1894: 22–23), *T. penduliflora* K. Schum. (1896: 225), and especially *T. ventricosa*.

The conservation status of *T. cameroonensis* was assessed using GeoCAT (Bachman et al. 2011) by estimating the extent of occurrence (EOO) and area of occupancy (AOO) from known localities. AOO was calculated using the standard 2 × 2 km grid recommended by the IUCN (IUCN Standards and Petitions Committee 2024). Observations on habitat quality, population size, and threats were used to propose a preliminary conservation assessment. Maps were generated using QGIS v.3.34 (QGIS Development Team 2023).

The taxonomic status of *Domkeocarpa pendula* Markgr. was investigated. The lectotype, *Deistel 137*, was examined. The specimen bears flowers but lacks fruit. Comparisons of our material with *D. pendula* therefore relied primarily on the original description, and herbarium specimens from the region of Mount Cameroon.

Terminology follows Leeuwenberg (1976, 1991), Endress et al. (2019), and Beentje (2021).

## Taxonomy

### Tabernaemontana cameroonensis

Taxon classificationPlantaeGentianalesApocynaceae

Ver.-Lib. & Libalah nom. nov.

1BFBC03F-805F-5CF4-8AE1-271A9D7418C8

urn:lsid:ipni.org:names:77484452-1

Figs 1, 2

≡ Domkeocarpa pendula Markgr., Notizbl. Bot. Gart. Berlin-Dahlem 15: 421. 1941.

#### Type.

Cameroon. South-West Region: Buea, s.d., *Deistel 137* (lectotype: **Z** Z-000001421[designated by Leeuwenberg (1991)]; isolectotype: **P** P00413452).

#### Nomenclatural note.

*Domkeocarpa* Markgr. is a synonym of *Tabernaemontana* (Leeuwenberg 1976; Endress et al. 2019). The type of *D. pendula*, together with newly collected flowering and fruiting material from Cameroon and São Tomé, demonstrate that this is distinct from *T. ventricosa* Hochst. ex A.DC.

*Domkeocarpa pendula* cannot be transferred to *Tabernaemontana* because the validly published name, *T. pendula* Woodson (Ann. Missouri Bot. Gard. 27: 534. 1940), already exists. Consequently, the replacement name *Tabernaemontana cameroonensis* Ver.-Lib. & Libalah is proposed here. *T. cameroonensis* is typified by the type of *D. pendula* (ICN Art. 7.4; Turland et al. 2018).

#### Diagnosis.

*Tabernaemontana cameroonensis* Ver.-Lib. & Libalah is most similar to *T. ventricosa* but differs by its cap-like flower buds; corolla lobes twisted to the left, with strongly undulate margins; and compact, syncarpous fruit with non-divergent, densely verrucose, irregularly ridged mericarps (vs. flower buds not cap-like; corolla lobes with weakly undulate margins; and fruit fused only at the base, with spreading, obliquely ellipsoid mericarps bearing only two faint lateral ridges).

#### Description.

**Figure 1.**
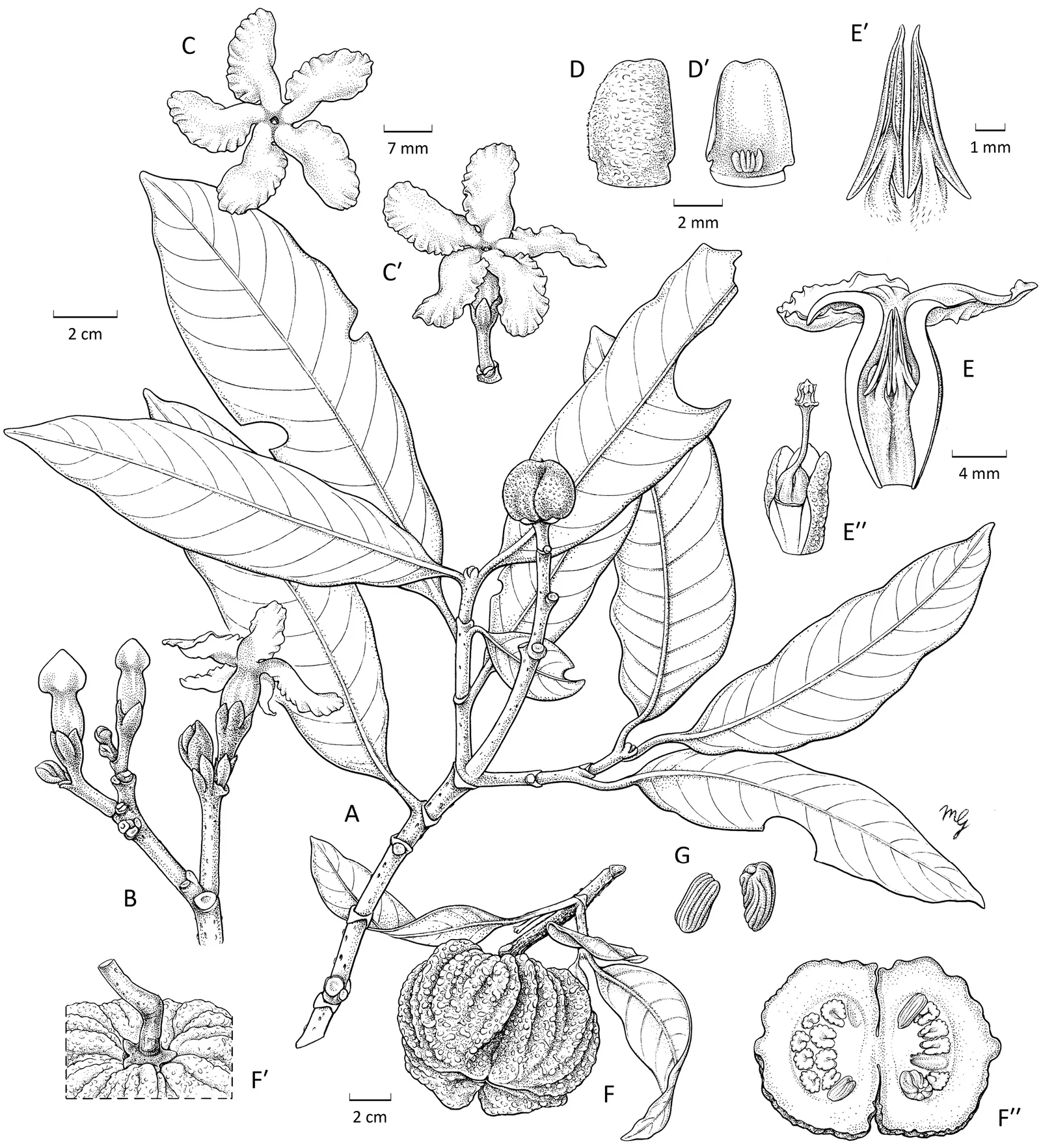
*Tabernaemontana cameroonensis*. **A**. Fruiting branch showing opposite leaves; **B**. Inflorescence with developing flower buds; **C**. Flower, front view; **C'**. Flower, lateral view; **D**. Calyx lobe, inner surface showing dense median rows of colleters; **D'**. Calyx lobe, outer surface; **E**. Corolla dissected longitudinally, showing the insertion of stamens and indumentum within the tube. E'. Detail of stamens in the dissected corolla tube; **E"**. Pistil; **F**. Young syncarp showing the compact, deeply bilobed fruit with a densely verrucose surface and numerous irregular longitudinal ridges; **F'**. Apical region of the syncarp showing pedicel insertion; **F"**. Transverse section of the syncarp showing the thick fleshy pericarp and seeds surrounded by fleshy arilloid tissue; **G**. Seeds. Drawn from *M. Libalah* 323 (**B–E"**) and *M. Libalah & M.C. Veranso-Libalah* 339, 341 (**A, F, G**) by Maria Geyer.

**Figure 2.**
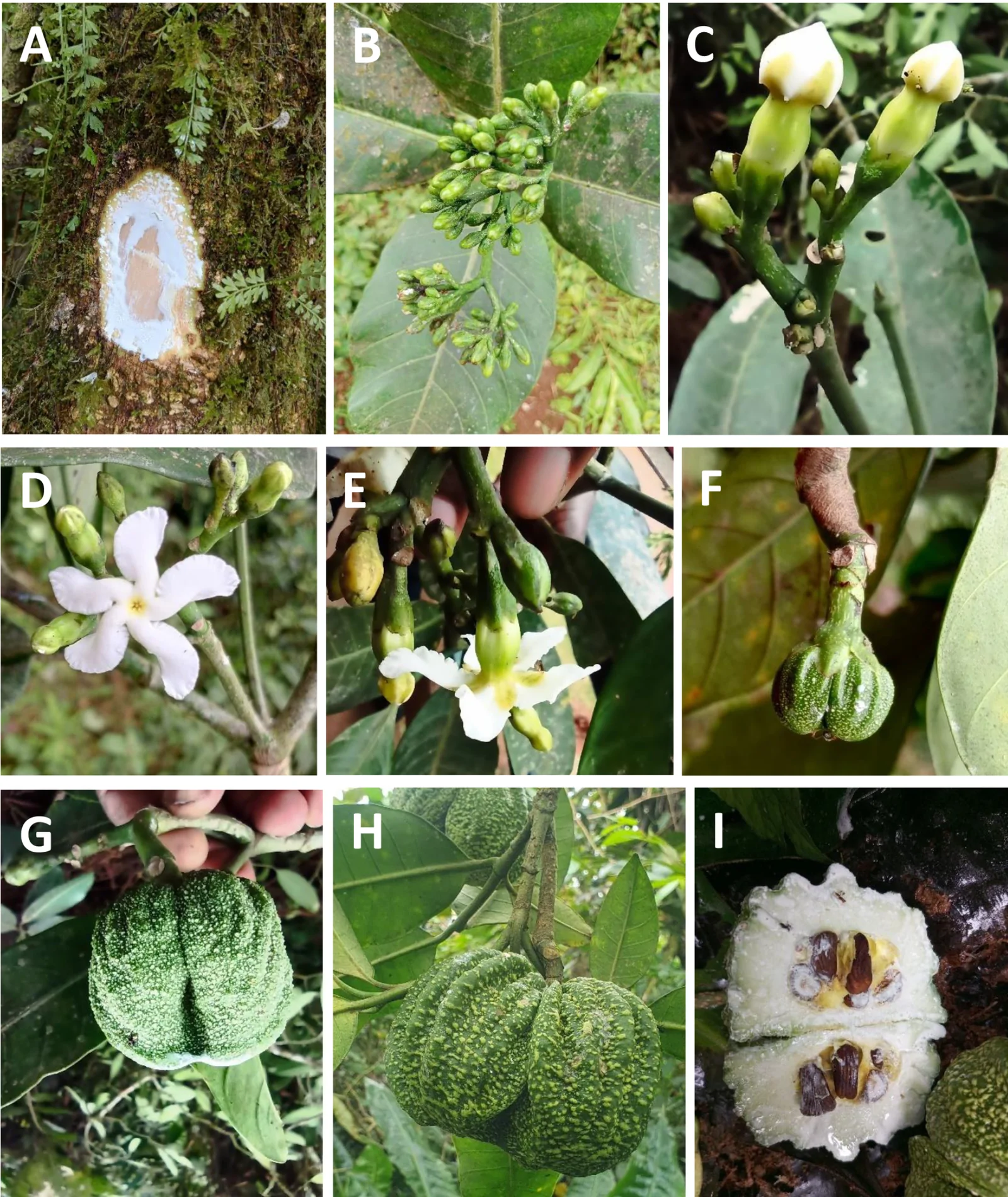
*Tabernaemontana cameroonensis***A**. Bark of runk slashed showing abundant white latex; **B**. Inflorescence with many developing buds; **C**. Buds showing characteristic clavate form and cap-like, rounded to truncate apex; **D**. Frontal view of flower showing spreading lobes twisted to the right and yellow throat of tube; **E**. Flower viewed obliquely from below, showing the strongly undulating margins of lobesand pale yellow throat; **F**. Immature syncarp; **G**. Immature fruit with verrucose surface and longitudinal ridges already visible; **H**. Mature slightly bilobed syncarp with verrucose surface and irregular longitudinal ridges; **I**. Transvere section of fruit showing thick fleshy pericarp and brown seeds with yellow–orange arils. Photographs by M.B. Libalah (**A–E**; *M. Libalah* 323, 322) and M.C. Veranso-Libalah (**F–I**; *M. Libalah & M.C. Veranso-Libalah* 339, 341).

Small tree 6–8 m tall; exuding abundant white latex when cut (Fig. 1A). Branchlets glabrous; young axes with conspicuous pale lenticels. ***Leaves*** opposite; pairs equal or slightly unequal; blade 8–23 × 3.8–8.5 cm, elliptic, base obtuse, apex acute, chartaceous to subcoriaceous; adaxial surface dark green, abaxial surface pale glaucous-green; midrib prominent above; secondary veins 12–16 pairs, strongly raised beneath; tertiary venation inconspicuous; petiole 8–18 mm long, glabrous. ***Inflorescences*** terminal, usually paired, often appearing lateral, umbelliform cymes, 15- to 70-flowered, glabrous; ***peduncle*** 6–22 cm long; bracts early deciduous; ***pedicel*** 7–14 mm long, glabrous. **Calyx lobes** greenish; 2.5–4 × 2.5–4 mm, rounded to broadly ovate; outer surface minutely papillate to very sparsely puberulent; margins thin, ± hyaline, ciliate or ciliolate at the base, otherwise entire; inner surface at base with 1–2 dense rows of closely spaced colleters (c. 6–20 per lobe) arranged along the median line, otherwise glabrous (Fig. 1D–D'). ***Buds*** narrowly clavate below, expanding distally into a compact, cap-like, broadly ovoid to subglobose tip; apex thickened, somewhat fleshy, obtuse to broadly rounded or slightly truncate, smooth and glabrous, the corolla lobes meeting without visible separation (Figs 1B, 2B, 2C). ***Corolla*** externally pale green at base, becoming white distally; throat pale yellow; ***tube*** 8–16 × c. 4 mm, subcylindrical, slender in the lower part, gradually widened above the middle, not twisted; inner surface sparsely pubescent from the insertion of the stamens to the throat; ***lobes*** 14–22 × 4–6 mm, lanceolate, distinctly twisted to left, spreading to slightly reflexed; margins strongly undulate, with fine cilia restricted to one margin (left in aestivation), otherwise glabrous; apices rounded to obtuse (Figs 1B, 1C, 2D, 2E). ***Stamens*** included, inserted 3.5–4.5 mm above base of corolla tube; anthers c. 6 mm long, glabrous. ***Pistil*** 9–15 mm long, glabrous; ovary cylindrical, c. 1.5 mm long, consisting of two free carpels; style c. 6 mm long, linear; pistil head c. 2 mm long, with a basal annular thickening. ***Fruit*** a compact, deeply bilobed syncarp, 8–14 × 5–15 cm, formed by two tightly appressed, non-divergent mericarps; mericarps broadly ovoid to subglobose, markedly thickened; surface densely verrucose to coarsely granulose, bearing numerous pale, lenticel-like protuberances and irregular, unevenly developed longitudinal ridges, giving the fruit a strongly lobulate appearance; green throughout development and at maturity; pericarp thick, fleshy and white (Figs 1F–F", 2F–I). ***Seeds*** several, dark brown to black, partially enclosed by yellow to orange arils.

#### Etymology.

The specific epithet *cameroonensis* is derived from Cameroon, the English spelling of the country name (French: Cameroun), the country where the type locality is situated.

#### Distribution and ecology.

**Figure 3.**
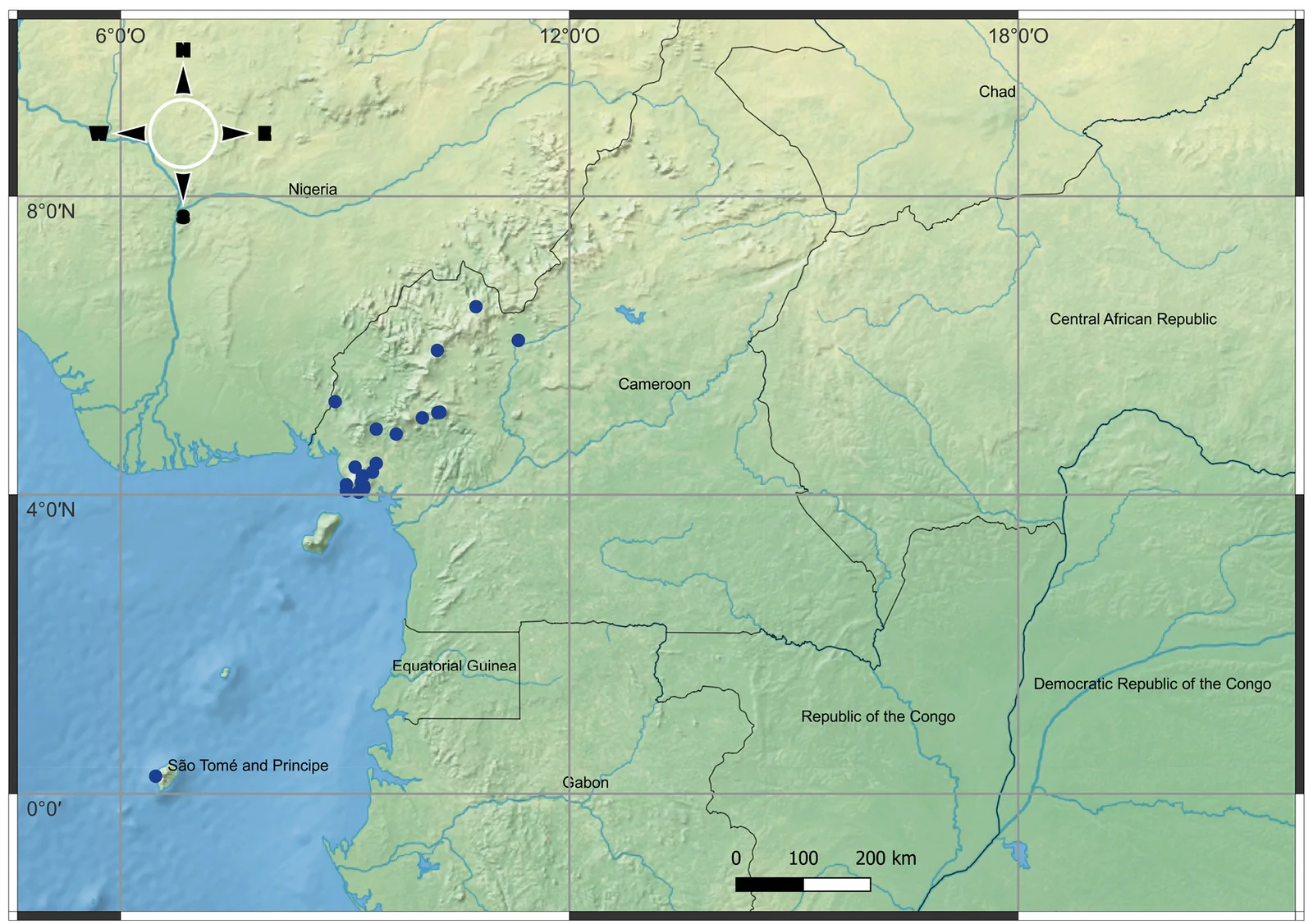
Known distribution of *Tabernaemontana cameroonensis* in Cameroon and São Tomé. In Cameroon, occurrence records are from the Adamawa, Northwest, West, and Southwest Regions.

Current evidence shows that *T. cameroonensis* occurs in the Lower Guinean subcentre of endemism in western Cameroon and on São Tomé in the Gulf of Guinea (White 1979). In Cameroon, records are concentrated in the western part of the country, principally in the Northwest, West, and Southwest administrative Regions, with an additional record from the Adamawa Region (Fig. 3). The species has been recorded from several highland areas, including the Bamenda Highlands, Mount Bana, Mount Kupe, the Rumpi Hills, Mount Cameroon, and Bandoumkassa. In Cameroon, *T. cameroonensis* occurs mainly in submontane to montane forest at 550–2,200 m a.s.l., with the São Tomé collection occurring near sea level at 10 m a.s.l.

The species occurs in humid evergreen forest, including forest margins and areas with an open or disturbed canopy, as well as in disturbed forest remnants, occasionally on steep slopes.

The distribution of *T. cameroonensis* suggests it is a characteristic component of the montane and submontane forests of the Cameroon Volcanic Line. Its occurrence on São Tomé indicates a broader biogeographic link among Gulf of Guinea montane floras. Despite its wide range, the species appears to be uncommon locally and is known from few collections.

#### Phenology.

Flowering mainly March–May; fruiting June–October.

#### Conservation status.

Many herbarium specimens previously identified as *T. ventricosa* are now referred to *T. cameroonensis*, which is known from 31 collections in western and southwestern Cameroon and on São Tomé (Fig. 3). Its extent of occurrence (EOO) is estimated at 73,200 km^2^ and its area of occupancy (AOO) at 84 km^2^, using the IUCN-recommended 2 × 2 km grid. Many of its habitats are under pressure from agriculture and development, leading to degradation and fragmentation of the forests.

Consequently, *T. cameroonensis* is provisionally assessed as **Vulnerable [VU B2ab(iii)]** (IUCN Standards and Petitions Committee 2024). Further field surveys are needed to assess population sizes and trends across its range.

#### Additional specimens examined.

**Cameroon. Adamawa Region** • Mbor village, 06°04'N, 011°19'E, 20 Feb 1963, *Brunt 979* (K, YA), *Brunt 980* (K, YA). — **Northwest Region** • Tabenken Mountain, 06°31'N, 010°45'E, 14 Nov 1974, *Letouzey 13233* (NHN, P, YA) • Mt Nshele (= Mba Mfeu), 05°56'N, 010°14'E, 12 Aug 1975, *Letouzey 14246* (NHN, P, YA). — **West Region** • Mt Bana, 05°02'N, 010°02'E, Jan 1939, *Jacques-Félix 2988* (P, YA) • Bandoumkassa, 05°06.20'N, 010°14.30'E, 6 Jun 2022, *22-ENSM-3-6* (BR, M, YA) • 2 Apr 2024, *M. Libalah 322* (BR, FR, M, YA), *M. Libalah 323* (BR, BRLU, FR, K, M, YA) • 8 Jul 2025, *M. Libalah & M.C. Veranso-Libalah 339* (BR, FR, M, YA), *M. Libalah & M.C. Veranso-Libalah 341* 8 Jul 2025, *M. Libalah & M.C. Veranso-Libalah 341* (BR, FR, M, YA) • 16 Aug 2025, *M. Libalah 347* (BR, FR, M, YA). — **Southwest Region** • Mount Cameroon, Buea, 04°02'N, 009°11'E, 17 Jan 1926, *Dunlap 220* (K) • Mount Cameroon, 18 Feb 1927, *Dalziel 8346* (K) • Jan 1929, *Maitland 273* (K) • Mar 1929, *Maitland 506* (K), Mar 1929, *Maitland 528* (K) • May 1929, *Maitland 676* (K) • Feb 1930, *Maitland s.n. 67* (K) • 14 Apr 1947, *Dundas 15335* (K) • between Hut 1 and Buea, 04°10'N, 009°13'E, 7 Feb 1962, *Breteler, de Wilde, Leeuwenberg & Letouzey 284* (NHN, YA) • near Hut 1 above Buea, 04°18'N, 009°22'E, 1 Oct 1983, *Thomas 2621* (K, MO, P) • above Batoke, 04°08'N, 009°01'E, 29 Dec 1983, *Thomas 2848* (BR, K, MO) • above Koto village, 04°03'N, 009°01'E, 6 Mar 1985, *Thomas 4486* (BR, K, MO, P, YA) • near Efolofo, 04°22'N, 009°08'E, 1 Jun 1976, *Letouzey 15061* (NHN, P, YA) • 4 Mar 1988, *Nemba, Thomas, Mambo & Crosby 936* (MO, YA) • 8 Dec 1993, *Faucher, Etuge & Furneaux 21* (K) • 9 Dec 1993, *Cable & Mukete 491* (K) • 16 Mar 1995, *Cable et al. 1628* (K). Mt Kupe, 28 May 1996, *Ryan 347* (K) • Nyasoso, 24 Jun 1996, *Etuge, Gupta, Katende & Assefa 2393* (K) • Mt Kupe, 11 Jul 1996, *Cable et al. 3790* (K) • Rumpi Hills, Mar 1984, *Thomas 3293* (K, MO, P) • Korup National Park, 27 Apr 1996, *Cheek 8243* (K, YA). — **São Tomé And Príncipe. São Tomé** • Lembá District, between Bindá and Juliana de Sousa, near Ponta Furada, 00°14'N, 006°28'E, 10 Dec 1978, *Groenendijk 133* (BR, NHN, S).

**Figure 4.**
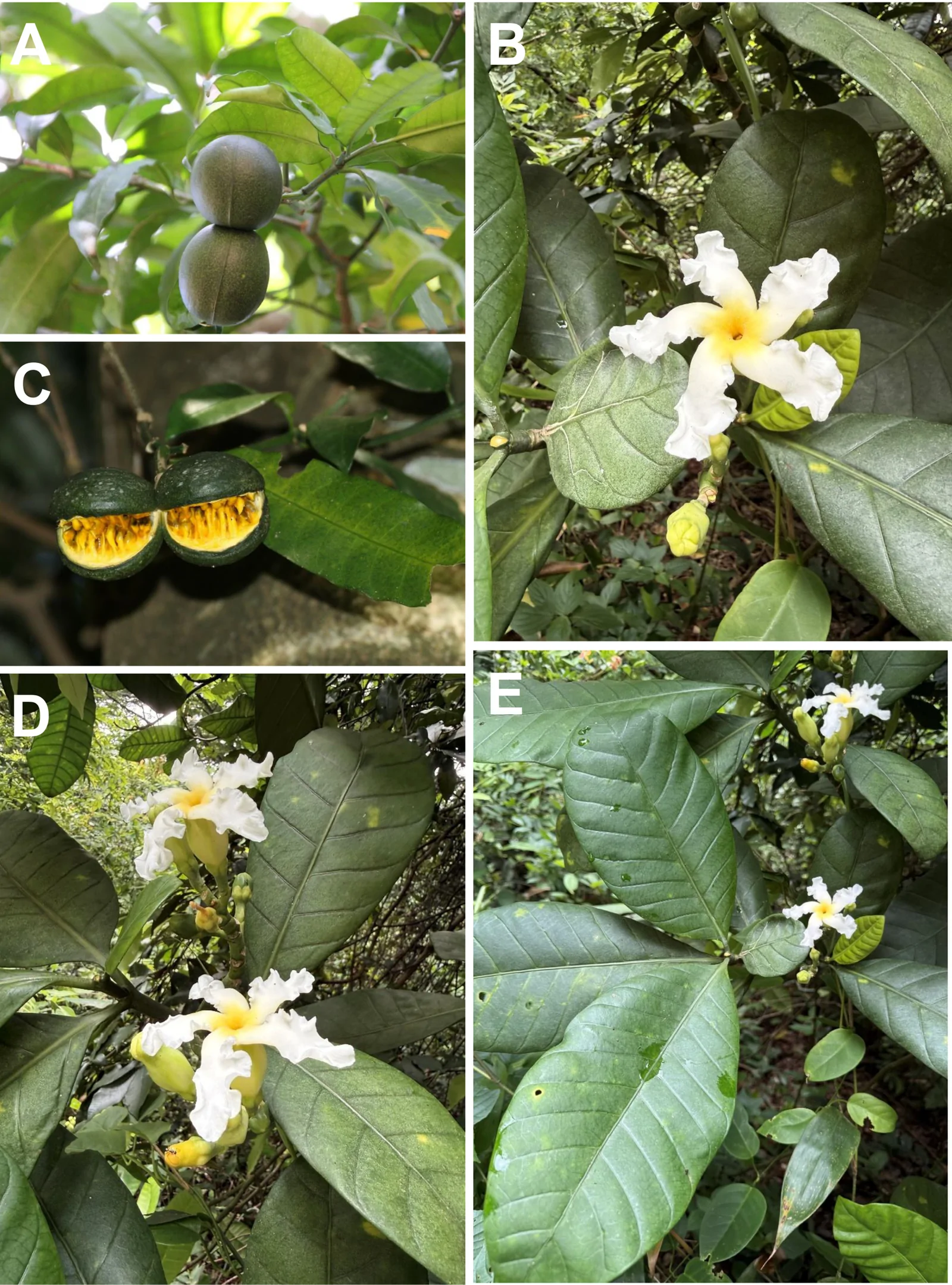
*Tabernaemontana ventricosa* Hochst. ex A.DC. from South Africa. **A**. Mature fruit showing paired divergent and non-connate mericarps with a comparatively smooth surface than *T. cameroonensis* in Fig. 2 G, H; **B**. Dehiscent fruit opened along the ventral suture, showing the orange pericarp and seeds; **C**. Flower, frontal view; **D**. Flowering branch; **E**. Flowering shoot with leaves and flower. (Photographs: **A**. Gray; **B**. Sharon Louw; **C–E**. Babette Köhler).

## Discussion

### Taxonomic implications for the *Tabernaemontana ventricosa* complex

The species treated here was long included as part of the widespread *T. ventricosa*, but it differs markedly from the original southern African concept of that species.

The circumscription of *T. ventricosa* was later expanded by Leeuwenberg (1976) to include morphologically similar material from eastern, central, and southern tropical Africa. There, *T. ventricosa* was characterized by obliquely ellipsoid fruit with two faint lateral ridges and a weakly sculptured surface. These features match southern and eastern African material but differ markedly from many Caneroonian specimens that Leeuwenburg included in *T. ventricosa*. These Cameroonian collections closely match *Domkeocarpa pendula* from the Mount Cameroon region (Markgraf 1941). Leeuwenberg (1976) reduced *D. pendula* to synonymy under *T. ventricosa*, thereby incorporating Cameroonian material into his broad concept of *T. ventricosa*.

The lectotype (*Deistel 137*) bears only flowers and lacks fruit. The remaining original material, which may have included fruit, was probably destroyed [by the carpet-bombing by the Allies] in Berlin during World War II. Markgraf (1941) provided a clear account of a compact syncarp of two deeply bilobed, non-divergent mericarps. This is consistent with our newly collected Cameroonian material.

Following Leeuwenberg (1976), collections from Cameroon and São Tomé were identified as *T. ventricosa*. These specimens consistently differ from the southern and eastern African material in both their flowers and fruit (details in Table 1).

**Table 1.** Comparison of *T. cameroonensis* with the morphologically similar African species *T. psorocarpa*, *T. brachyantha*, *T. pachysiphon*, and *T. ventricosa*.

**Character**	***T. cameroonensis***	***T. psorocarpa***	***T. brachyantha***	***T. pachysiphon***	***T. ventricosa***
**Habit**	Small tree (to 10 m)	Liana, climbing (to 20 m)	Tree (to 20 m)	Shrub or tree (to 15 m)	Shrub or tree (to 15 m)
**Leaves (shape)**	Elliptic	Narrowly elliptic	Broadly elliptic to oblong–elliptic	Elliptic	Elliptic
**Leaf size (cm)**	8–23 × 3.8–8.5	4–15(–20) × 1–6	(5–)7–55 × 4–30	10–50 × 5–26	4–27 × 2–12
**Secondary veins**	12–16 pairs	4–10 pairs	8–15 pairs	7–15 pairs	6–12 pairs
**Inflorescence**	Large terminal, umbelliform cymes (15 to 70-flowered)	Few-flowered (7–10)	Large, many-flowered, branched	Lax, few-flowered	Contracted, many-flowered
**Calyx outer surface**	Minutely papillate to sparsely puberulent	Glabrous	Glabrous	Glabrous (margins ciliate)	Glabrous (margins ciliate)
**Calyx colleters (inner)**	1–2 dense medial rows (6–20)	Absent	3–5 rows, less organised	Present, not in dense medial rows	Present
**Bud shape**	Clavate with cap-like rounded apex	Ovoid to subglobose	Broadly ovoid	Ovoid	Broadly ovoid
**Corolla tube length (mm)**	8–16	10–15	12–18	18–42	10–27
**Corolla tube shape**	Subcylindric (inflated above middle)	Subcylindric	Pentagonal	Pentagonal	Cylindric, non-angular
**Tube torsion**	Not twisted	Twisted (0.25–0.5 turn)	Slightly twisted	Slightly twisted	Slightly twisted
**Throat colour**	Distinctly yellow	White or cream	White to yellowish	Yellowish	Yellow
**Corolla lobes**	Strongly undulate, asymmetrical	Weakly undulate, falcate	Moderately undulate	Slightly undulate	Falcate, weakly undulate
**Lobe symmetry**	Asymmetrical)	Oblique	Subsymmetric	Subsymmetric	Oblique
**Lobe ciliation**	Unilateral along left margin	Basal or weak	Weak or absent	Absent	Basal auricle on left side
**Fruit**	Compact, slightly bilobed syncarp	Two divergent follicles	Two slightly divergent follicles	Two divergent follicles	Two free, slightly divergent follicles
**Mericarp shape**	Broadly ovoid–subglobose	Oblong–ellipsoid	Subglobose, slightly flattened	Subglobose	Oblong–ellipsoid to ellipsoid
**Fruit size (cm)**	8–14 × 5–15	5–6 × 2–3.5	4–5 × 4–6	7–15 × 6–14	6–10 × 4–10
**Fruit surface**	Densely verrucose to coarsely granulose	Bossellate, uneven	Smooth	Smooth	Slightly verrucose
**Longitudinal ridges**	Numerous, irregular, prominent	Weak crests only	Absent	Absent	Two weak lateral crests
**Fruit wall thickness**	Very thick	10–12 mm	5–15 mm	23–40 mm	7–13 mm
**Distribution**	Cameroon and São Tomé and Príncipe	Nigeria–Gabon	W–Central Africa	W–Central to E Africa	East and South Africa

We conclude that the plants formerly identified as *T. ventricosa* in Cameroon and São Tomé represent a distinct species corresponding to *D. pendula*. We recognise this species as *T. cameroonensis* Ver.-Lib. & Libalah, nom. nov. *Tabernaemontana ventricosa* is restricted to southern and eastern Africa.

### Comparison with *Tabernaemontana* species from Cameroon and São Tomé

Although *T. cameroonensis* was previously included in *T. ventricosa*, it is readily distinguished from the other species included in the present treatment (Table 1; Key below). The key below is adapted from Leeuwenberg (1991), with modifications from our investigations. *Tabernaemontana ventricosa* s.str. is included for comparison.

### Key to the species of *Tabernaemontana* in Cameroon and São Tomé (including *T. ventricosa* s.str. for comparison)

**Table:** 

1	Plants lianescent or sarmentose shrubs	**2**
–	Plants erect shrubs or trees	**5**
2	Flowers usually opening at night; corolla tube elongate; fruits green or glaucous at maturity	**3**
–	Flowers opening during the day; corolla tube relatively short; mature fruits pale bluish-green or yellow to orange	**4**
3	Calyx lobes without colleters, 1.2–2.4 times as long as wide; corolla tube 25–60 mm long; peduncle 3–50 mm long	***T. eglandulosa***
–	Calyx lobes with 3–10 basal colleters, 1–1.5 times as long as wide; corolla tube 45–86 mm long; peduncle 3–7 mm long	***T. glandulosa***
4	Calyx lobes suborbicular to broadly ovate, as broad as or broader than long; corolla tube white, 13–18 mm long; corolla lobes 13–20 mm long, pubescent only along the left margin; fruits narrowly ovoid, longitudinally ridged but otherwise smooth, yellow to orange	***T. fragrans***
–	Calyx lobes elliptic to ovate, longer than broad; corolla tube pale green, 9–14 mm long; corolla lobes 7–17 mm long, puberulous basally on both sides; fruits broadly ovoid, irregularly bumpy, pale bluish-green	***T. psorocarpa***
5	Corolla tube distinctly pentagonal or 5-angular	**6**
–	Corolla tube cylindrical to subcylindrical	**7**
6	Corolla tube 12–18 mm long; throat densely pubescent; corolla lobes moderately undulate; mericarps depressed-subglobose to transversely ellipsoid	***T. brachyantha***
–	Corolla tube robust, 18–42 mm long, slightly twisted at base; throat glabrous; mericarps pale glaucous, thick-walled	***T. pachysiphon***
7	Peduncle absent or up to 3 mm long; corolla tube 10–13 mm long, distinctly inflated around the anthers; flowers often pendulous; mericarps subglobose to transversely ellipsoid	***T. penduliflora***
–	Peduncle generally longer; corolla tube not distinctly inflated around the anthers	**8**
8	Corolla tube strongly twisted, approximately one complete turn; stamens inserted 16–22 mm above its base; inflorescences several-flowered; mericarps pale green, obliquely subglobose	***T. contorta***
–	Corolla tube straight or twisted less than one complete turn; stamens inserted lower in the tube	**9**
9	Mature flower bud with a conspicuously enlarged ovoid head much broader than the corolla tube; stamens inserted 11–15 mm above its base; mericarps very large, 10–20 cm long	***T. stapfiana***
–	Mature flower bud with head not conspicuously broader than the corolla tube; mericarps generally smaller	**10**
10	Corolla tube 34–100 mm long, 8–20 times as long as the calyx lobes, distinctly twisted; corolla pubescent within below the stamens; leaves large and coriaceous	***T. crassa***
–	Corolla tube 8–34 mm long, or if 70–120 mm long then very slender and associated with yellow to orange mericarps	**11**
11	Corolla tube very slender, 70–120 mm long; petioles 5–15 mm long; mericarps yellow to orange, non-subglobose	***T. hallei***
–	Corolla tube 8–34 mm long	**12**
12	Buds acute at apex; peduncle slender, 10–20(–40) mm long; inflorescence lax, 3–11-flowered; corolla tube 9–15 mm long	***T. inconspicua***
–	Buds rounded, truncate, ovoid or broadly ovoid at apex	**13**
13	Buds cap-like, rounded to truncate at apex; calyx lobes with 1–2 dense median rows of colleters; corolla tube straight, 8–16 mm long, with a conspicuous yellow throat; corolla lobes strongly undulate and asymmetrical; fruits forming a compact, deeply bilobed syncarp, mericarps tightly appressed and indehiscent at maturity, broadly ovoid to subglobose, densely verrucose to coarsely granulose with numerous irregular longitudinal ridges	***T. cameroonensis***
–	Buds ovoid to broadly ovoid; fruits composed of two distinct mericarps, free or only basally united	**14**
14	Corolla tube slender, 19–34 mm long, 6–10 times as long as the calyx; inflorescence lax, few- to many-flowered	***T. stenosiphon***
–	Corolla tube 10–27 mm long, 2–5.5 times as long as the calyx; inflorescence contracted, many-flowered; fruits dehiscent along the ventral suture at maturity, the mericarps becoming widely divergent; mericarps obliquely ellipsoid, smooth to weakly sculptured, with two faint longitudinal ridges; aril orange	***T. ventricosa* s.str**.

## Supplementary Material

XML Treatment for Tabernaemontana cameroonensis

## Additional information

### Artificial Intelligence (AI) use

The authors accept full responsibility for the content of the manuscript, including the disclosure of any use of AI.

No AI tools were used in the preparation of this manuscript.

### Author ORCIDs

M.C. Veranso-Libalah https://orcid.org/0000-0001-7847-1740

M.B. Libalah https://orcid.org/0000-0001-8848-8001

O. Yemdji https://orcid.org/0009-0007-3274-6324

A. Gröger https://orcid.org/0009-0001-8521-2795

B. Sonké https://orcid.org/0000-0002-4310-3603

G. Kadereit https://orcid.org/0000-0003-0094-8769

### Data availability

All of the data that support the findings of this study are available in the main text.
